# Circulating Fibrocytes: Cellular Mediators of Tissue Fibrosis

**DOI:** 10.3390/ijms27020557

**Published:** 2026-01-06

**Authors:** Xinya Guo, Jianyu Lu, Yiyao Du, Zhaofan Xia, Shizhao Ji

**Affiliations:** Department of Burn Surgery, The First Affiliated Hospital of Naval Medical University, Shanghai 200433, China; guoxinya0724@163.com (X.G.); 18796246278@163.com (J.L.); duyiyao1127@126.com (Y.D.)

**Keywords:** circulating fibrocytes, fibrosis

## Abstract

Fibrosis is a pathological condition resulting from an excessive tissue response during the repair process, often affecting various tissues such as the skin, organs, and joints, posing a significant threat to global health. Researchers have made substantial efforts to explore the endogenous mechanisms underlying fibrosis in recent years and have developed several therapeutic strategies to block this process. Historically, research on fibrotic diseases has focused on identifying highly relevant therapeutic targets and developing effective antifibrotic drugs. However, due to the complexity of the mechanisms of fibrosis and its effector cells, the effectiveness of antifibrotic therapies remains limited. With the advancement of high-throughput omics technologies and machine learning tools, we now have a clearer understanding of cellular heterogeneity, intercellular interactions, and the specific roles of cells in various biological processes. This enables tracking the trajectory of different cell types during the fibrotic process, facilitating early identification and discovery of new targets for fibrosis treatment, and conducting more precise targeted research. Supported by these novel technologies, numerous studies have revealed that, in addition to normal fibroblasts, a group of bone marrow–derived fibrocytes also contributes to the fibrosis of both parenchymal and non-parenchymal organs and tissues. Circulating fibrocytes are hematopoietic-derived cells that are recruited to injury sites during injury, disease, and aging, acting as participants in inflammation and tissue repair, and directly or indirectly promoting fibrosis in various tissues throughout the body. This review summarizes the general characteristics of circulating fibrocytes, the molecular mechanisms involved in their recruitment to different tissues, the process of their differentiation into fibroblasts, their potential roles in various diseases, and the latest research developments in this field. Given the key role of circulating fibrocytes in fibrosis across multiple tissues, they may serve as promising targets for the development of novel antifibrotic therapies.

## 1. Introduction

Fibrosis is a pathological condition resulting from an excessive tissue response during the repair process. It often affects various tissues such as the skin, organs, and joints. Although fibrosis itself is not a disease, it poses a serious threat to global health. In developed countries, the mortality rate associated with fibrotic diseases can reach as high as 45% [1,2]. According to statistics from the World Health Organization, five out of the top ten leading causes of death globally in 2019 were directly related to fibrosis, and these diseases have been showing an increasing trend year by year. In China, the incidence of fibrosis is also gradually rising. Fibrotic diseases associated with various systems, including the cardiovascular, respiratory, and digestive systems, have become major public health concerns [3]. In recent years, researchers worldwide have made significant efforts to explore the endogenous mechanisms associated with fibrosis and have tested numerous therapeutic strategies aimed at blocking this process. Previous research on fibrotic diseases has primarily focused on identifying therapeutic targets closely related to fibrosis and developing effective antifibrotic drugs. However, due to the complexity of the mechanisms underlying fibrosis and its effector cells, the application of antifibrotic therapies has proven to be less effective. As a result, there is an urgent need to discover new approaches to address fibrosis.

With the advancement of high-throughput omics technologies and machine learning tools, we are now able to gain a clearer understanding of cellular heterogeneity, intercellular interactions, and the specific functions of cells in various biological processes. These technologies enable us to track the trajectory of different cell types during the fibrotic process, allowing for the early identification and discovery of new therapeutic targets for fibrosis, and facilitating more precise targeted research. The innovation of these methods has provided stronger support for the role of circulating fibrocytes in the fibrotic process, further clarifying their key role in fibrosis.

First, circulating fibrocytes are derived from the bone marrow and can rapidly respond to tissue injury signals. They migrate to the damaged site via the bloodstream, and this migratory ability makes them key participants in the repair process. Second, during tissue repair, circulating fibrocytes secrete a variety of cytokines that promote the inflammatory response, further attracting other immune cells to the injured area and contributing to the formation of a complex pathological environment [4]. Additionally, these cells can differentiate into fibroblasts within the damaged tissue, promoting the synthesis of collagen and other extracellular matrix components, thereby exacerbating the progression of fibrosis [5]. Circulating fibrocytes were first described in 1994 by R. Bucala [6]. These hematopoietic-derived cells are recruited to wound sites following tissue injury and during disease. Acting as participants in inflammation and tissue repair, they directly or indirectly promote fibrosis in systemic tissues [7].

With the discovery of this unique cell population, researchers have increasingly characterized fibrocytes, examining their morphological features, in vitro growth characteristics, biological functions in vivo, as well as their potential use as biomarkers and therapeutic targets in various diseases. These findings indicate that fibrocytes play a crucial role in chronic inflammation, wound healing, tissue remodeling, and the process of fibrosis.

This review summarizes the general characteristics of circulating fibrocytes, the molecular mechanisms by which they are recruited to different tissues, the process of their differentiation into fibroblasts, their potential roles in various diseases, and the latest research developments in this field. Given the key role of circulating fibrocytes in fibrosis across multiple tissues, they may serve as promising targets for the development of novel therapeutic strategies for fibrosis.

## 2. Functional Characteristics of Circulating Fibrocytes

### 2.1. Origin of Circulating Fibrocytes

The term “fibrocyte” was first used to describe cells that combine characteristics of fibroblasts with those of leukocytes, platelets, and erythrocytes. These cells have been reported to display fibroblast-like ECM-associated signals (including collagen-related proteins and transcripts) together with hematopoietic features such as CD34 [8]. The derivation and differentiation of circulating fibrocytes is a complex and finely regulated biological process, involving multiple cell types, signaling pathways, and influences from the microenvironment.

Researchers initially believed that these fibrocytes originated from fibroblasts migrating from the surrounding tissue into the wound bed. However, studies have shown that these cells can be observed within just two days after injury, and their numbers are unexpectedly high. The timing and quantity of their appearance led researchers to conclude, “The formation of this cell population is not solely due to the slow migration of cells from adjacent connective tissue; it is likely that they also migrate from the peripheral blood.” The concept that fibroblast-like cells found in the wound bed originate from peripheral blood dates back nearly a century. Since then, researchers have conducted more in-depth investigations into this process. Multiple lines of evidence support a bone marrow/hematopoietic contribution to fibrocyte-like cells in injury models; however, the precise trajectories and boundaries relative to monocyte-derived macrophage states are context-dependent and continue to be refined. Under appropriate conditions, hematopoietic stem cells can differentiate into various types of progenitor cells, particularly monocyte progenitors. Within the bone marrow, these monocyte progenitors undergo a series of stringent developmental processes and eventually mature into monocytes. Mature monocytes are then released into the peripheral blood, becoming an important component of the immune system and playing a crucial role in specific physiological and pathological conditions. In the event of trauma or tissue injury, monocytes are induced by various signals from the microenvironment, causing monocytes in the peripheral blood to rapidly migrate to the damaged area, where they participate in local immune responses and repair processes. Moreover, these signals not only promote the migration of monocytes but also trigger their transformation into circulating fibrocytes, which are capable of producing large amounts of collagen and other extracellular matrix components, thereby supporting tissue regeneration and repair. According to previous studies, fibrocytes account for 0.1–0.5% of the circulating non-erythrocytic cells. Although their numbers are low, they respond very rapidly and may represent a unique intermediate stage between bone marrow–derived progenitor cells, capable of differentiating into the monocyte lineage, and mature stromal cells under certain conditions. Newly discovered evidence, including the expression of CD11a, CD11b, CD32, and CD64, supports the hypothesis that fibrocytes originate from monocytes. However, some evidence contradicts the notion of fibrocyte monocyte origin. During the process of monocytes maturing into pro-monocytes, CD34 expression is absent. Additionally, most researchers consider fibrocytes to be CD14− cells, whereas monocytes express the CD14 marker. This discrepancy may, however, be part of a series of marker changes that occur during cell differentiation.

In addition, mesenchymal stem cells (MSCs) in the bone marrow also possess multidirectional differentiation potential, with the ability to differentiate into cells related to bone, cartilage, muscle, and adipose tissue [9]. However, they do not appear to be precursor cells of fibrocytes, as circulating or tissue-resident MSCs and mesenchymal cells do not express CD34 (a hematopoietic progenitor cell marker) or CD45 (a common leukocyte marker), both of which are considered key markers for identifying fibrocytes. In contrast, hematopoietic stem cells express the CD34 marker, which is one of the strongest pieces of evidence supporting the origin of fibrocytes from hematopoietic progenitor cells. Furthermore, fibrocytes not only express myeloid cell markers such as CD13, but in a model of carotid artery grafts implanted in sheep, circulating leukocytes labeled with carboxyfluorescein diacetate and succinimidyl esters were observed to gradually migrate to the injured tissue, acquiring a fibrotic phenotype (CD34, CD45, vimentin, and α-SMA) in the newly formed tissue [10]. These studies further support the hypothesis that fibrocytes originate from hematopoietic cells.

Based on previous studies, fibrocytes are not only structurally closely related to bone marrow–derived cells but also functionally play a significant role in tissue injury repair. A substantial body of evidence supports the hypothesis that fibrocytes originate from the bone marrow. They are derived from the myeloid lineage, arising from hematopoietic stem cells, and represent an intermediate stage in the differentiation of monocyte lineage progenitors into mature stromal cells. As research in this field progresses, the origin of fibrocytes and their role in various diseases will be more comprehensively understood, providing new insights and directions for future therapeutic strategies.

### 2.2. Markers of Circulating Fibrocytes

A major challenge in fibrocyte research is that the currently used markers are informative but not fully specific. CD45 is a pan-leukocyte marker and therefore cannot discriminate fibrocytes from monocytes/macrophages, whereas CD34 expression may vary with maturation stage, tissue context, and experimental conditions [11]. Importantly, intracellular type I collagen protein positivity in circulating cells should not be interpreted as definitive evidence of de novo collagen biosynthesis, because collagen can be acquired through uptake mechanisms and staining readouts may be influenced by technical factors (e.g., extracellular matrix adherence, permeabilization-dependent signal, or antibody cross-reactivity) [12]. Accordingly, fibrocyte identification should rely on multi-marker phenotyping and, when possible, be strengthened by functional assays (e.g., ECM deposition) and transcriptomic evidence (e.g., COL1A1/2 transcripts at single-cell resolution), rather than on CD45/CD34/collagen signals alone. Recent scRNA-seq studies further indicate that populations historically labeled as fibrocytes can comprise multiple transcriptionally distinct states with substantial overlap with myeloid compartments. These data strengthen the view that fibrocyte assignment is more robust when anchored to integrated phenotypic, functional, and transcriptomic criteria rather than a limited marker set [13]. To clarify the phenotypic and functional overlap between fibrocytes and closely related cell populations, we provide a comparative summary of commonly used markers and key functions in Table 1.

The lack of specific markers for fibrocytes has posed significant challenges in studying these cells in vivo. In previous studies, researchers typically distinguished fibrocytes from other cell types based on the expression of cell surface markers associated with hematopoietic stem cells (CD34), leukocytes (CD45, and leukocyte-specific protein-1), monocytes (CD11a, CD11b, CD32, and CD64), and fibroblasts (collagen type I, collagen type III, fibronectin, and vimentin) [26,27]. Although fibrocytes express some markers of monocytes and macrophages (such as F4/80, CD68), they do not fully match these cell types. Markers such as CD14 and CD16 are often reduced compared with circulating monocytes, but their expression can be variable across studies and differentiation states. Additionally, fibrocytes produce type I and type III collagen—markers not typically found in monocytes or macrophages—and express CD34 [28,29,30]. However, collagen protein detection alone is insufficient to infer biosynthetic capacity, because collagen may be internalized from the extracellular environment and assay-dependent artifacts can contribute to apparent positivity. Furthermore, fibrocytes differ from dendritic cells, B cells, and T cells in terms of their marker expression. Fibrocytes share substantial phenotypic overlap with monocytes/macrophages, including expression of multiple myeloid-associated markers in many settings; therefore, fibrocytes are most robustly interpreted as a fibroblast-like program emerging within a hematopoietic compartment rather than as a population separable by a single marker [29]. In addition, fibrocytes also express chemokine receptors CCR2, CCR3, CCR5, CCR7, CXCR4, CXCR6, and CX3CR1, the host defense Fc receptors for immunoglobulin G (CD32 and CD64), lymphocyte activation antigen presentation and co-stimulatory molecules (major histocompatibility complex [MHC] class I and II/CD80 and CD86), as well as cell surface enzymes (CD10 and CD13) [31].

In most previous studies, the identification of fibrocytes primarily relied on a combination of different markers. Initially, the recognition of circulating fibrocytes was mainly based on the co-expression of CD34 or CD45 with type I collagen or type III collagen [32,33,34]. Subsequently, with the development of multi-parameter flow cytometry, fibrocyte-like cells can be phenotyped with greater granularity using broader marker panels. Nevertheless, classification remains constrained by marker non-specificity and assay-dependent variability, and is strengthened when combined with orthogonal readouts such as collagen gene expression (e.g., COL1A1/2), procollagen detection, or functional ECM deposition assays. Andreas Weigert et al. more precisely identified fibrocytes using markers such as CD45, Col1, CD44, CD163, CCR5, CD33, S100A8, CD162, CXCR3, and CCR2 [14]. This multi-marker flow cytometric approach allows for a more comprehensive depiction of the functions and changes in fibrocytes across different tissues. In practice, rigorous flow-cytometric identification requires appropriate gating and controls and should be interpreted together with orthogonal evidence such as transcriptomic or functional readouts.

However, during the maturation process in the hematopoietic system, the expression of surface markers on fibrocytes often changes. These alterations, to some extent, complicate the identification of these intermediate cells, making it more challenging to differentiate them from other cell populations [5,35]. Consequently, researchers have employed various strategies to study the role of fibrocytes in injury and disease models. In addition to the circulating cell labeling technique, common methods include immunohistochemistry, transgenic mouse models, and bone marrow chimeric mouse models. Immunohistochemistry is a commonly used method, but it has certain limitations. When identifying fibrocytes, researchers often use Col1 immunostaining as evidence of Col1 expression. However, immunohistochemistry can only show whether a protein is present in the cell but cannot distinguish whether this collagen is produced by the cell or absorbed from the surrounding environment. Fibrocytes, in particular, possess a high capacity for collagen uptake, which further complicates this distinction [12]. Therefore, collagen-based staining should be interpreted as supportive rather than definitive, and fibrocyte assignment should not rest on collagen positivity alone. Transgenic mice, by integrating exogenous DNA into the genome, enable the transcription of specific genes to label and trace cell lineages. However, this model may introduce non-specific labeling due to cell plasticity or non-target cells expressing Cre recombinase. In fibrocyte research, chimeric mouse models are often used to trace hematopoietic-derived fibrocytes. By performing lethal irradiation to deplete the host’s bone marrow cells and reconstituting them with bone marrow cells from genetically distinct donors, researchers can distinguish between host- and donor-derived cells. However, since MSCs contained in the bone marrow may reside in tissues like the lungs, this model may overestimate the contribution of hematopoietic-derived cells to tissue fibrosis [36]. To more accurately label fibrocytes, researchers have utilized various markers such as CD45, Col1, and CD44, combined with multiple transgenic mouse models. However, the labeling efficiency of multi-transgenic models is low, and the breeding schemes are more complex. Additionally, researchers have employed approaches like chimeric models and transcriptomic analysis. In chimeric models, the circulatory systems of two organisms are surgically connected to study the contribution of fibrocytes. Transcriptomic analysis, using techniques such as single-cell RNA sequencing (scRNA-Seq), has revealed the heterogeneity of fibrocyte subpopulations and their gene expression profiles [13]. These research tools provide new perspectives and approaches for a deeper understanding of fibrocytes in tissue fibrosis and repair.

Overall, the emergence of new technologies has shown tremendous potential in cell labeling, providing more reliable tools for studying the functions and characteristics of fibrocytes. With the continuous advancement of genomics and single-cell technologies, we are likely to discover new markers that will enable more precise identification and classification of fibrocytes. This will allow for a more comprehensive depiction of fibrocyte behavior under different physiological and pathological conditions and offer deeper insights into their roles in tissue repair and disease progression.

### 2.3. Recruitment of Circulating Fibrocytes

Fibrocytes or their precursor cells first enter the bloodstream from the bone marrow as pre-differentiated collagen-producing cells. These cells then travel through the peripheral blood to the injured tissue, where they complete their differentiation and promote tissue repair. Although fibrocytes or fibrocyte precursors account for only 0.1–0.5% of the non-red blood cells circulating in the blood, approximately 10% of them migrate to the wound site to participate in the healing process. Studies have shown that the peak migration of fibrocytes into the blood and wound site occurs on days 3 and 5 after injury, respectively. During this period, local inflammation persists while repair processes such as granulation tissue formation begin.

The migration of fibrocytes is guided by physical and chemical signals in the environment, particularly the action of chemokines [37]. Fibrocytes express a variety of chemokine receptors on their surface, including CCR2, CCR3, CCR5, CCR7, CXCR4, CXCR6, and CX3CR1 [26,29,38,39,40]. These receptors bind to their respective chemokines, collectively guiding fibrocytes to the site of injury. Notably, the interaction between the CCR7 receptor and CCL21 plays a crucial role in the migration of fibrocytes. Studies have shown that endothelial cells at the wound site express CCL21, providing a chemotactic signal for the migration of fibrocytes [29]. Therefore, the interaction between CCR7 and CCL21 is one of the key mechanisms by which fibrocytes migrate to the early wound site. Further research has demonstrated that CCR7+ fibrocytes, through their binding with CCL21, recruit fibrocytes in tissues such as the kidney, promoting the occurrence of fibrosis. For example, in a mouse model of kidney fibrosis, CCR7+ fibrocytes help induce renal fibrosis following ureteral obstruction [27]. Additionally, other studies indicate that the interaction between CXCL12 and CXCR4 plays a critical role in pulmonary fibrosis. CXCR4+ fibrocytes show a strong response to CXCL12, and the concentration gradient of CXCL12 in the lungs may guide CXCR4+ fibrocytes to the fibrotic regions [26]. Thus, although the roles of chemokines and their receptors vary across different tissues, they play a key guiding role in fibrocyte migration. The interactions between different chemokines and receptors exert distinct functions in various pathological processes.

Fibrocytes also express a variety of molecules related to cell adhesion and intercellular interactions, such as CD9, CD11a, CD11b, CD11c, CD18, CD43, CD164, LSP1, CD34, CD29, CD44, CD81, CD49, and CD81 [31]. Although all of these receptors have been identified on the surface of fibrocytes, they are not all expressed simultaneously. Research by Phillips et al. has shown that at least two distinct fibrocyte subpopulations are present in a pulmonary model (CD45+ Col I+ CXCR4+ and CD45+ Col I+ CCR7+) [26]. Similarly, Sakai et al. described the presence of CCR7-CXCR4- and CCR2+ fibrocytes in a mouse model of renal fibrosis [27].

Furthermore, the interaction between the extracellular matrix (ECM) and its degrading enzymes can also promote the migration of circulating fibrocytes. Studies have shown that when fibrocytes migrate through the alveolar epithelial basement membrane, the proteolytic activities of MMP-2 and MMP-9 are important regulatory factors, while the activity of MMP-8 is closely associated with the promotion of the cell migration process [41,42]. These enzymes degrade the basement membrane and the ECM, providing physical pathways for fibrocyte migration, thereby enhancing their migration efficiency and directionality.

**Figure 1:**
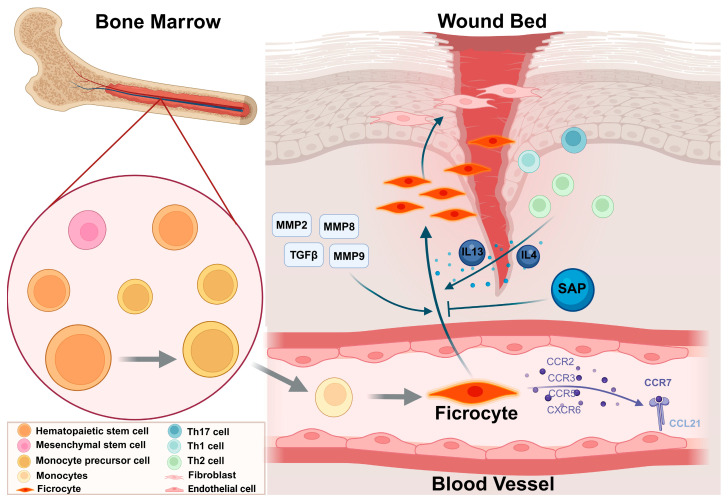
Migration and recruitment of circulating fibrocytes.

Fibrocytes play a crucial role in the wound healing process, migrating to the injury site in response to the interaction between chemokines and their receptors, thereby participating in the repair response. In addition, adhesion molecules on the cell surface and the degradation of the extracellular matrix provide further support for fibrocyte migration, the specific process is shown in Figure 1. Future studies may further elucidate the specific roles of various chemokines and receptors in different diseases, particularly in pathological processes such as fibrosis, and explore their potential clinical applications.

## 3. Regulatory Factors of Circulating Fibrocyte Differentiation

Identifying the factors that drive fibrocyte differentiation is as important as determining the location and origin of fibrocytes. Recent studies on the factors regulating circulating fibrocyte differentiation have further enriched our understanding, particularly in the exploration of regulatory mechanisms in fibrotic diseases. As research progresses, investigators have expanded their focus beyond traditional differentiation-promoting and differentiation-inhibiting factors, revealing more complex regulatory networks.

In recent years, the relationship between the immune system and the process of fibrosis has become a research hotspot. Increasing evidence suggests that the differentiation of fibrocytes is largely dependent on CD4(+) T cells, and the activation context of T cells determines whether fibrocyte development is supported or blocked [43]. CD4(+) T cells can differentiate into helper T cells, such as Th1, Th2, or Th17 cells, which are characterized and distinguished by the cytokines they secrete and their specific functions [44]. Fibrosis-promoting cytokines, such as IL-4 and IL-13 produced by Th2 cells, can promote the differentiation of fibrocytes from peripheral blood mononuclear cells [45], whereas anti-fibrotic cytokines like IL-12 and IFN-γ inhibit fibrocyte differentiation.

During the process of fibrosis, the normal tissue structure is replaced by excessive ECM. Circulating fibrocytes may participate in these fibrotic responses through recruitment from the circulatory system. This process involves complex interactions among various cytokines and signaling pathways, with key fibrosis-promoting factors such as Transforming Growth Factor-beta 1 (TGF-β1), Interleukin-13 (IL-13), and Interleukin-4 (IL-4). These factors promote the differentiation of circulating fibrocytes into fibroblasts and subsequent tissue fibrosis by activating the SMAD signaling pathway and the JAK/STAT signaling pathway [29,46,47]. Studies have shown that TGF-β1 is secreted in its latent form and binds with latent-associated peptides, which then interact with latent TGF-β binding proteins (LTBPs) to form ECM [42]. This process involves various proteases, including MMP-9, which cleave latent-associated peptides, LTBPs, or ECM proteins to facilitate the release of TGF-β. Research by Chiang et al. indicated that R1R2 is a peptide that reduces the proteolytic activity of MMP-9, preventing the release of TGF-β and thereby inhibiting the differentiation of myofibroblasts mediated by MMP-9. Studies by Jingyin Yan et al. have shown that the activation of bone marrow–derived fibrocytes is mediated by the Smad3 signaling pathway [48]. However, the knockout of Smad3 does not completely eliminate fibrocyte activation and ECM protein expression in vivo, suggesting that TGF-β1 may participate in fibrocyte activation through other pathways. Other studies have demonstrated that elevated serum amyloid P (SAP) levels can inhibit pulmonary fibrosis by suppressing fibrocyte differentiation, without affecting the expression of TGF-β1 [49]. These studies suggest that although TGF-β1 plays a key role in fibrocyte activation and tissue fibrosis, its mechanism of action may be more complex, involving interactions among various proteins and signaling pathways.

Research has shown that SAP is an inhibitor of fibrocyte differentiation, widely present at wound sites and in the blood, and it can inhibit the extramedullary differentiation of fibrocytes. In diseases associated with high circulating levels of TGF-β, TGF-β acts as a strong activator of fibrocyte differentiation, often accompanied by a decrease in SAP levels, thus promoting fibrocyte differentiation in the bloodstream. At the same time, studies have found that as the injured tissue heals during the repair process, SAP levels gradually decrease. This change may create conditions for infiltrating leukocytes to transform into a fibrocyte phenotype. These observations suggest that, although the fibrocyte differentiation process primarily occurs in the bone marrow, fibrocytes may also differentiate in the bloodstream or at the wound site. Based on these findings, a possible model has been proposed: fibrocyte precursors may exist in the bone marrow or blood, completing differentiation before migrating to the injured tissue, or these precursor cells may directly migrate to the injury site and undergo differentiation in the local environment.

Additionally, researchers have revealed that the differentiation of fibrocytes is influenced by the tumor microenvironment. For example, chronic inflammation and cytokine signaling within tumors can drive the further differentiation of these cells into myofibroblasts, which exhibit stronger pro-tumorigenic activity [50]. Numerous studies have shown that fibrocytes with a spindle-shaped morphology and CD34 expression are observed around the ductal structures in the breast, including areas of epithelial hyperplasia, ductal carcinoma in situ (DCIS), fibroadenomas, and phyllodes tumors [51]. However, local tumor factors may influence the further differentiation of fibrocytes into myofibroblasts, characterized by the loss of CD34 and the upregulation of αSMA expression. This transition is more commonly observed in locally invasive and malignant tumors [52,53]. Fibrocytes are typically identified by co-localization of CD45 and COL1 markers. Studies suggest that fibrocyte-like cells participate in tumor progression and immune evasion in various cancer types through multiple mechanisms.

In conclusion, the differentiation of fibrocytes is regulated by multiple factors, with the immune system, cytokines, and the tumor microenvironment interacting to determine the differentiation process. By thoroughly investigating the relationships between these factors and signaling pathways, we not only deepen our understanding of the mechanisms underlying fibrosis but also provide a rich theoretical foundation for the development of future therapeutic strategies targeting circulating fibrocytes.

## 4. Circulating Fibrocytes in Tissue Fibrosis

**Figure 2:**
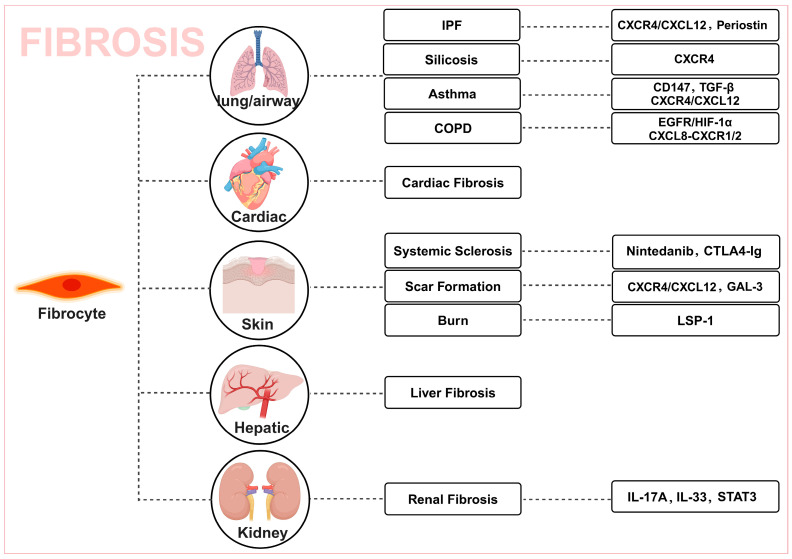
The influence and regulatory factors of circulating fibrocytes in various organs.

Tissue regeneration and repair processes are crucial, with abnormal remodeling being a key feature of many chronic fibrotic diseases. In fibrosis of organs such as the liver, lungs, kidneys, and skin [38], the origin, migration, and differentiation behavior of circulating fibrocytes in the local microenvironment are regulated by multiple signaling pathways, significantly influencing the progression and prognosis of fibrosis [11]. Figure 2 illustrates the influence and regulatory factors of circulating fibroblasts in various organs. Therefore, an in-depth study of the role and molecular mechanisms of circulating fibrocytes in fibrosis across different tissues is of great clinical significance for the development of novel therapeutic strategies targeting fibrotic diseases.

### 4.1. Lung/Airway

Common fibrotic diseases of the lungs and airways include Idiopathic Pulmonary Fibrosis (IPF), sarcoidosis, pneumoconiosis, connective tissue disease-related pulmonary lesions (such as those associated with rheumatoid arthritis and systemic sclerosis), hypersensitivity pneumonitis (HP), asthma, and Chronic Obstructive Pulmonary Disease (COPD). Research has shown that bone marrow–derived fibrocytes play a central role as pro-fibrotic effector cells in lung and airway fibrosis diseases through multiple mechanisms, including proliferation, differentiation, extracellular matrix production, inflammation maintenance, and interactions with other cells.

IPF several previous studies have observed higher levels of circulating fibrocytes in the lungs and peripheral blood compared to healthy controls, suggesting that circulating fibrocytes may have prognostic significance [34]. Research has analyzed and compared several commonly observed blood biomarkers in IPF, including chemokines (IL-8, CCL18), matrix metalloproteinases (MMP-1 and MMP-7), growth factors (IGBPs family), circulating T cells, and fibrocytes, but none of these biomarkers have been proven to be specific for IPF or predict disease progression (such as a decline in lung function, acute exacerbations, or the risk of death) [54]. It has been suggested that an increase in circulating fibrocytes (>5%) in IPF patients is associated with early mortality [55]. However, the exact role of fibrocytes in the disease still requires independent validation in prospective clinical cohorts. Iain D. Stewart et al. conducted a study and found that elevated levels of circulating fibrocytes were associated with the overall mortality of IPF, particularly when the level of circulating leukocytes was ≥2.2% (circulating leukocytes ≥ 2.2%). However, the increase in fibrocyte levels does not represent the severity of IPF, indicating that their value as a prognostic marker is not comprehensive [56]. Despite this, many studies focused on the role of circulating fibrocytes in IPF treatment are still actively progressing, and researchers have identified several potentially meaningful pathways and targets that may have a positive impact on the treatment and prognosis of IPF.

In recent years, drugs such as pirfenidone and nintedanib have been found to inhibit the release of pro-inflammatory and pro-fibrotic mediators, suppress fibrocyte migration and differentiation, and reduce extracellular matrix deposition, showing considerable therapeutic potential for IPF [57,58,59]. It is known that CXCR4 may drive fibrocyte migration and promote tissue fibrosis by binding to its specific ligand, CXCL12 [60,61]. Additionally, both CXCR4 and its ligand CXCL12 are highly upregulated in IPF lung tissue [62]. Consequently, researchers have focused on targeting CXCR4 to inhibit the differentiation of circulating fibrocytes. The i-body AD-114 has been identified as specifically binding CXCR4 and inhibiting fibrocyte accumulation and differentiation [63]. Furthermore, studies have shown that fibrocytes induce fibrosis by secreting periostin and other soluble factors that promote the differentiation of myofibroblasts. This mechanism could represent a promising direction for future research aimed at improving IPF treatment [64].

In studies on silicosis, fibrocytes play a crucial role, especially in the early stages of the disease [65]. When silica particles enter the lungs, they activate alveolar macrophages, which release a variety of inflammatory mediators that further stimulate fibrocyte proliferation and differentiation. Under the influence of these signals, fibrocytes proliferate and produce excessive collagen and other extracellular matrix components, leading to pulmonary fibrosis and structural damage to lung tissue. In recent years, research targeting fibrocytes to slow the progression of silicosis has been actively pursued. Chao Li et al. found that in a mouse model, dioscin effectively reduced fibrocyte recruitment, protected epithelial cells from crystalline silica-induced damage, inhibited TGF-β/Smad3 signaling, and prevented fibrocyte activation, thereby significantly delaying the progression of diagnosed silicosis [66]. Qixian Sun et al. discovered that in a silicosis mouse model, AMD3100 could reduce the recruitment of circulating fibrocytes to the lungs by blocking CXCR4, thus slowing lung inflammation and fibrosis [67]. Furthermore, CD137, an effective co-stimulatory molecule for T cell activation, was shown by Chao Li et al. to play a role in crystalline silica-induced pulmonary fibrosis. Blocking its downstream 4-1BB signaling pathway not only significantly reduced fibrocyte recruitment to the lungs but also alleviated fibrosis by modulating the local immune environment, thus mitigating lung damage and providing a potential therapeutic strategy [68].

The airway remodeling and fibrosis induced by asthma and COPD are to some extent controllable, making this a highly valuable area of research. Studies have shown that when asthma patients are exposed to allergens, circulating fibrocytes can migrate to the bronchial mucosa, where they differentiate into cells capable of producing ECM. Additionally, the abundance of fibrocytes is correlated with the thickness of the basement membrane [69], and its thickening serves as a hallmark of airway remodeling in asthma patients. Furthermore, research has found that the number of circulating fibrocytes is positively correlated with both the decline in lung function and the severity of asthma [70,71], suggesting that fibrocytes play a detrimental role in the progression of asthma. Recent studies in an ovalbumin (OVA)-induced asthma mouse model have indicated that CD147 regulates the proliferation, differentiation, and chemotactic function of fibrocytes through its influence on the TGF-β1 and CXCL12/CXCR4 signaling pathways. Inhibition of CD147 reduces the accumulation and fibrotic activity of fibrocytes in the lungs, thereby decreasing airway inflammation and remodeling. This could be a promising target for future asthma therapies [72].

Unlike asthma, research on COPD primarily focuses on the role of fibrocytes during acute exacerbations. Although the overall number of circulating fibrocytes does not significantly increase during the stable phase of COPD, there is a notable rise in fibrocyte numbers in the blood during acute exacerbations. This suggests that fibrocytes play a role in the body’s repair process in response to acute inflammation or tissue damage. After the acute exacerbation resolves, approximately two months later, the fibrocyte levels gradually return to baseline [73]. Chun-Hua Wang and colleagues investigated the activation mechanisms of fibrocytes in COPD patients and found that the EGFR/HIF-1α axis promotes the activation of fibrocytes. They proposed the potential for developing new therapeutic strategies by intervening in this pathway [74]. Additionally, recent studies have highlighted a potential direct interaction between fibrocytes and CD8+ T cells in the bronchial regions of COPD patients, particularly in the distal airways. CD8+ T cells in COPD tissue promote the chemotaxis of fibrocytes via the CXCL8-CXCR1/2 axis. This interaction may be a key factor driving the progression of the disease [75].

Circulating fibrocytes play a central role in various fibrotic diseases of the lungs and airways, with their proliferation, differentiation, and interaction with the local microenvironment directly influencing the onset and progression of these diseases. Therapeutic strategies targeting these cells, particularly through the modulation of signaling pathways involved in fibrocyte migration and activation, have become a key focus of current research.

### 4.2. Cardiac

Cardiac fibrosis is a common pathological endpoint of diverse cardiovascular conditions and is closely associated with adverse remodeling, myocardial stiffening, impaired diastolic function, and the formation of an arrhythmogenic substrate [76]. In addition to resident cardiac fibroblasts and fibroblast progenitors, increasing evidence suggests that circulating fibrocytes contribute to cardiac fibrogenic remodeling [77]. Clinically, elevated circulating fibrocyte levels have been reported in patients with hypertension, heart failure, coronary artery disease, and atrial fibrillation. These increases have been detected in peripheral blood as well as in cardiac tissue specimens when compared with healthy controls [77,78]. A similar elevation has been described in aging-associated cardiac remodeling, supporting the concept that immunosenescence and chronic low-grade inflammation facilitate fibrocyte mobilization and recruitment to the heart [79]. Consistent with this notion, fibrocyte abundance has been linked to diffuse myocardial fibrosis in humans assessed by cardiac magnetic resonance–derived indices, extending potential clinical relevance to non-ischemic remodeling phenotypes [80].

Beyond these associations, several studies indicate potential biomarker value. Circulating fibrocyte abundance has been proposed as a surrogate of left atrial fibrosis burden and as a predictor of recurrence after catheter ablation in persistent atrial fibrillation, suggesting utility for risk stratification and post-procedural surveillance [78]. Elevated circulating fibrocytes have also been reported in unstable angina and were associated with adverse clinical outcomes, implying that fibrocyte-related signatures may reflect a broader inflammatory–fibrotic axis relevant to cardiovascular risk [81]. Nevertheless, the specificity of circulating fibrocytes as biomarkers of cardiac fibrosis remains uncertain, because fibrocyte expansion can be driven by systemic inflammation and may parallel fibrotic activity in other organs [76,77].

Mechanistic insight into fibrocyte involvement in cardiac fibrosis largely derives from experimental models of myocardial injury and pressure or volume overload. In these models, fibrocyte-like populations have been detected within injured myocardium and fibrotic regions, consistent with recruitment from the circulation and local accumulation during remodeling [82,83,84]. Early studies in ischemic cardiomyopathy provided evidence that bone marrow–derived fibroblast precursors can contribute to myocardial remodeling and dysfunction [83], while investigations in chronically failing hearts supported a measurable contribution of bone marrow–derived cells to collagen-producing populations [82]. In addition, chemokine-guided trafficking appears to be relevant in hypertensive and neurohormonal injury. CCR2 signaling was shown to regulate the accumulation of bone marrow–derived fibroblast precursors and to modulate angiotensin II–induced cardiac fibrosis [85]. Activation of the SDF-1 and CXCR4 axis has also been linked to enhanced fibrocyte recruitment and myocardial fibrosis in vivo [84]. At the same time, the magnitude and functional importance of hematopoietic contributions remain debated. Rigorous lineage analyses indicate that most infarct fibroblasts arise from epicardial lineages rather than from bone marrow, highlighting persistent controversy regarding cellular origins in post-infarction scarring [86].

Differences across studies likely reflect variation in marker-based definitions of fibrocytes and in the strategies used to distinguish fibrocytes from monocytes or macrophages that acquire ECM-associated signatures during injury responses. Divergent conclusions may also arise from the choice of injury model and the time window examined, because findings in the early inflammatory phase do not necessarily align with those observed during late scar maturation. Technical factors further contribute, including tissue digestion protocols, flow cytometry gating strategies, and immunostaining procedures, all of which can influence fibrocyte detection and quantification [76,77,86]. Accordingly, future work should prioritize standardized phenotyping with consensus marker panels and explicit exclusion strategies, together with causal perturbation approaches, to determine whether fibrocytes primarily function as direct ECM-producing effectors, paracrine inflammatory amplifiers, or both within cardiac fibrotic remodeling [76].

From a mechanistic perspective, trafficking of fibrocytes from bone marrow and peripheral blood into cardiac tissue represents a central step. In other fibrotic organs, the CXCL12 and CXCR4 axis is repeatedly implicated in fibrocyte mobilization and homing [77]. Similar gradients are likely engaged in the heart, supported by experimental evidence linking SDF-1 and CXCR4 activation to fibrocyte recruitment and myocardial fibrosis [76], as well as by patient data suggesting that circulating SDF-1 can promote fibrocyte migratory behavior in atrial fibrillation [78]. In parallel, inflammatory recruitment circuits mediated by CCL2 and CCR2, which are well established in cardiac fibrosis biology, may further facilitate the accumulation of hematopoietic fibroblast precursors in the myocardium [72,75,76,85]. After recruitment, fibrocytes can acquire fibroblast-like and myofibroblast-like features in response to pro-fibrotic cues such as TGF-β, mechanical stress, and inflammatory cytokines. This transition is associated with increased ECM production and enhanced expression of contractile markers such as alpha smooth muscle actin, thereby promoting interstitial and perivascular fibrosis [76]. Fibrocytes likely represent a spectrum of activation states rather than a single stable lineage, with state transitions influenced by local oxygen tension and macrophage-derived mediators [77]. In addition to direct ECM synthesis, fibrocytes may amplify fibrogenesis through paracrine output of pro-fibrotic and pro-inflammatory mediators, including pathways related to TGF-β1, interleukin 6, and tumor necrosis factor alpha. These signals can activate resident cardiac fibroblasts, shape macrophage polarization, and sustain chronic inflammatory circuits that favor fibrosis. Such immune–stromal coupling is particularly relevant to atrial remodeling, where inflammation–fibrosis interactions contribute to conduction heterogeneity and stabilization of an arrhythmogenic substrate [78].

Taken together, these findings position circulating fibrocytes as both candidate biomarkers and potential therapeutic targets in cardiac fibrosis. Therapeutic strategies could aim to limit maladaptive recruitment and homing, or to modulate inflammatory recruitment circuits that sustain fibrocyte accumulation and activation [84,85]. Consistent with this concept, CXCR4 antagonism has shown anti-fibrotic benefit in preclinical cardio-renal injury settings and has been reported to reduce cardiac fibrosis and improve cardiac performance in experimental models [87,88]. However, caution is warranted because fibrocyte-related pathways may also contribute to necessary repair processes, and broad suppression of recruitment or inflammation could impair wound healing after acute injury. Stage-specific targeting and careful patient stratification will therefore be essential to maximize benefit while minimizing unintended disruption of physiological repair programs [76].

### 4.3. Skin

Research on circulating fibrocytes in skin fibrosis primarily focuses on diseases related to systemic sclerosis (SSc) and scar formation. These cells play a key role in skin repair and fibrotic responses, and their characteristics, such as being more easily accessible on the skin surface, make them an important target for studying fibrosis mechanisms and potential therapeutic interventions. Compared to internal organs such as the heart, lungs, liver, and kidneys, skin fibrosis offers a more direct means of observation, providing a unique advantage in exploring the role and potential of circulating fibrocytes in the fibrotic process.

Circulating fibrocytes also play a significant role in the immunopathological mechanisms of SSc, where they interact with inflammatory cells and autoantibodies to promote the development of fibrosis. A recent study showed a significant positive correlation between circulating fibrocytes and skin thickness in patients with limited cutaneous systemic sclerosis (lcSSc). Researchers conducted modified Rodnan skin score (mRSS) and high-frequency ultrasound (US) assessments on the skin of 8 lcSSc patients and 5 healthy controls. The results indicated that the number of circulating fibrocytes was increased in lcSSc patients, suggesting that these cells may play a critical role in the progression of skin fibrosis and could serve as potential biomarkers [89].

This study is the first to demonstrate the direct regulatory role of circulating fibrocytes in lcSSc, providing a basis for future research and potential therapeutic targets. Furthermore, circulating fibrocytes play a crucial role in the immunopathological mechanisms of SSc. They participate in both innate and adaptive immune responses, interacting with inflammatory cells and autoantibodies such as antinuclear antibodies and anti-endothelial cell antibodies. Circulating fibrocytes can also express and secrete pro-inflammatory and pro-fibrotic cytokines, modulating local immune responses and enhancing the intensity of inflammation and fibrosis [90]. In recent years, there has been increasing research focused on targeting circulating fibrocytes to improve SSc. Maurizio Cutolo and colleagues found that Nintedanib inhibits the conversion of circulating fibrocytes to myofibroblasts and their pro-fibrotic activity in SSc patients, significantly downregulating the gene and protein expression of αSMA, S100A4, COL1, and FN in these cells [91]. Additionally, Nintedanib has been shown to exhibit anti-fibrotic activity in IPF, SSc-ILD, rheumatoid arthritis-ILD, hypersensitivity pneumonitis, and silicosis [57]. Another study indicated that CTLA4-Ig (Abatacept) significantly downregulated the expression of key fibrotic markers (such as αSMA and COL I) in circulating fibrocytes from SSc patients in vitro, with no significant effect on fibrocytes from healthy controls [92]. This suggests that circulating fibrocytes in SSc patients are more sensitive to CTLA4-Ig treatment, potentially due to higher expression levels of CD86 in these cells [93]. This finding provides a foundation for CTLA4-Ig as a novel early intervention therapy for SSc, particularly in targeting circulating fibrocytes to slow the progression of fibrosis.

Circulating fibrocytes also play a role in the scar formation process through similar mechanisms [94,95,96]. Following skin injury, these cells rapidly migrate to the damaged site, becoming part of the repair process. However, their excessive activation can lead to hyperproliferation of scar tissue, resulting in hypertrophic scars or keloids. One study evaluated the expression of circulating fibrocytes and the chemokine CXCL12 in keloid patients to elucidate their role in keloid formation. Flow cytometry analysis of blood samples from 10 keloid patients and 12 normal scar patients revealed that the number of circulating fibrocytes (CD45+, Col I+) in keloid patients (17.4 × 10^5^ cells/mL) and the absolute number of CXCR4+ fibrocytes were significantly higher than those in normal scar patients [97]. Additionally, the expression level of CXCL12 in keloid tissue (529.3 pg/mL) was also significantly upregulated. These studies suggest that the CXCR4/CXCL12 axis plays a key role in the formation and recurrence of keloids. Further research targeting this signaling pathway found that thymic stromal lymphopoietin (TSLP) in keloids acts not only as a potential initiator of collagen synthesis but also activates the CXCR4/CXCL12 axis, promoting the chemotaxis and local accumulation of fibrocytes, thereby exacerbating the pathological progression of keloid formation. This study revealed the significant pathological role of TSLP in keloid formation, suggesting it as a potential target for antifibrotic therapy in the future treatment of keloids [98,99]. Additionally, studies have shown that galectin-3 (GAL-3) in human keloid tissue is significantly positively correlated with the gene expression of type I collagen, indicating that GAL-3 may play an important role in the pathological formation of keloids [100].

In recent years, the role of circulating fibrocytes in scar formation caused by burns has become a new area of research. A study by Yang et al., through protein extraction and mass spectrometry analysis, found the presence of fibrocytes in burn wound healing sites, with their number closely associated with the severity of the burn and the formation of hypertrophic scars. Further analysis of the protein differences between fibrocytes and lymphocytes revealed that leukocyte-specific protein 1 (LSP-1) is one of the hallmark proteins of fibrocytes, which may serve as a new biomarker for assessing burn wound healing and predicting the formation of hypertrophic scars [101]. Additionally, research by Wang and Suda further explored the regulatory effect of fibrocytes on the activity of other cells, such as immune cells and fibroblasts. Their results suggested that fibrocytes, by modulating local immune responses and cytokine release, might play a key role in the excessive fibrotic response following burns, particularly in the formation of hypertrophic scars [96,102]. Rea et al., through murine experiments, further investigated the specific role of circulating hematopoietic lineage cells in burn wound healing. The study showed that the involvement of these cells is transient, mainly concentrated in the early stages of immune response and cell migration during wound healing [103]. Furthermore, Holland et al. confirmed the presence of fibrocytes in pediatric burn wounds, emphasizing that although the duration of their role in burn healing is relatively short, their impact on inflammation and early healing is crucial [104].

In summary, the role of circulating fibrocytes in burn wound healing is multifaceted and context-dependent. These cells contribute to the early stages of wound healing by engaging in immune responses and the synthesis of the extracellular matrix, thereby modulating the repair process. However, their involvement is both time-dependent and localized, and variations in circulating fibrocyte populations alone are insufficient to reliably predict the outcome of scar formation [16]. Current evidence suggests that circulating fibrocytes cannot yet be considered a reliable biomarker for the prognosis of skin fibrosis.

### 4.4. Hepatic

In the liver, fibrocytes have been increasingly linked to the coupling of inflammation and stromal remodeling during chronic injury. Although their assessment in hepatic tissue is complicated by phenotypic overlap with abundant infiltrating myeloid populations, convergent experimental and clinical observations support a disease-relevant role for fibrocytes in fibrogenic progression [40,105,106].

Experimental models of hepatic fibrogenesis indicate that fibrocytes can be mobilized from the bone marrow and recruited to the injured liver in response to fibrogenic insults [105,107,108]. Their trafficking is consistent with chemokine-guided migration, and studies of fibrogenic liver injury have implicated chemokine receptor programs that include CCR2 and CCR1 in regulating recruitment dynamics [107,109]. In carbon tetrachloride–induced injury, collagen-expressing fibrocyte-like populations show dynamic regulation of chemokine receptor expression, with age-associated increases in CCR2 and CCR7 together with induction of additional receptors, a pattern that may contribute to altered recruitment and inflammatory amplification in chronic disease settings [107]. These findings are consistent with a model in which fibrocytes behave as early, highly responsive circulating effectors that can shape the intrahepatic inflammatory milieu before, or in parallel with, maximal expansion of activated myofibroblasts.

Once within the liver microenvironment, fibrocytes have been proposed to contribute to fibrogenesis through two intertwined mechanisms. On one hand, they may adopt myofibroblast-like features and participate in extracellular matrix remodeling. On the other, they may act as paracrine amplifiers by releasing inflammatory and profibrotic mediators that influence immune–stromal crosstalk and promote hepatic stellate cell activation [6,105]. Importantly, the quantitative contribution of bone marrow–derived fibrocytes to hepatic collagen production remains debated. While several transplantation and tracing studies support the presence of bone marrow–derived collagen-expressing cells in fibrotic liver [105,107,108], a collagen reporter recipient model concluded that bone marrow–derived cells contribute only minimally to collagen production in toxic and cholestatic fibrosis [110]. Consistent with this view, systematic analyses of fibrogenic myofibroblast subsets in mice indicate that activated stellate cells and activated portal fibroblasts account for most collagen-producing myofibroblasts, whereas bone marrow–derived populations contribute a relatively small fraction in commonly used models [111,112]. Conversely, measurable involvement of CD34-positive fibrocytes has been described in chronic cholestatic contexts, suggesting that etiology, timing, and methodological sensitivity can substantially influence attribution [108,109]. Together, the current evidence supports a context-dependent model in which fibrocytes are consistently recruited during liver injury but variably contribute to structural matrix deposition across etiologies and stages.

Beyond the question of collagen source, several datasets support a biologically meaningful role for fibrocytes as inflammatory modulators in hepatic fibrosis. In a thioacetamide-induced model using bone marrow–restricted HSV thymidine kinase expression to enable valganciclovir-mediated depletion, removal of bone marrow–derived fibrocytes reduced hepatic hydroxyproline content and lowered serum alanine aminotransferase, consistent with attenuated matrix accumulation and hepatocyte injury [113]. Notably, depletion did not significantly reduce the number of hepatic myofibroblasts or the expression of major profibrotic drivers at the analyzed time point, whereas interleukin 1 beta levels were reduced, supporting the interpretation that fibrocytes can promote fibrogenic progression by shaping local inflammatory signaling rather than serving as a dominant collagen-producing population in this setting [113]. This framework aligns with the broader view that fibrocytes exhibit mixed immune and stromal functions and that their influence may be most apparent during phases of active recruitment, when inflammatory programs can set the trajectory of stellate cell activation and matrix remodeling [40,105].

Human observations further suggest that circulating fibrocytes may reflect hepatic fibrotic burden. In chronic hepatitis C cohorts, the proportion of peripheral blood fibrocytes increased with fibrosis stage and correlated with histological severity and liver stiffness measurements, supporting their potential utility as a minimally invasive disease-associated biomarker and an indirect readout of ongoing recruitment and activation [114]. Nevertheless, translation into a robust clinical tool will require standardized phenotyping and harmonized flow cytometry strategies, given the overlap between fibrocytes and other circulating myeloid subsets under systemic inflammatory conditions [40,106].

From a therapeutic perspective, fibrocytes are attractive targets because they sit upstream of immune recruitment–driven stromal activation loops. Serum amyloid P is an endogenous inhibitor of fibrocyte differentiation, and mechanistic work has linked this effect to Fc receptor signaling [115,116]. In liver fibrosis models, recombinant serum amyloid P, also known as PTX2, has been reported to attenuate fibrogenesis by limiting fibrocyte recruitment and suppressing hepatic stellate cell activation, thereby providing a translational anchor for targeting the fibrocyte axis while simultaneously dampening resident fibrogenic programs [117]. Overall, future work should prioritize etiology-stratified and stage-resolved quantification of fibrocyte contributions, improved harmonization of identification strategies in tissue and blood, and longitudinal human validation to clarify whether circulating fibrocytes improve risk prediction or treatment-response monitoring in chronic liver diseases [109,110,111,112,114].

### 4.5. Kidney

Circulating fibrocytes play a multifaceted and crucial role in the onset and progression of renal fibrosis by participating in inflammation, ECM deposition, and the accumulation of fibrotic cells, thereby regulating several key stages of the fibrotic process [118,119]. Studies have shown that the number of circulating fibrocytes is significantly elevated in various renal fibrosis models and correlates with the severity of renal fibrosis. In patients with lupus nephritis (LN), increased levels of circulating fibrocytes are closely associated with the progression of interstitial renal fibrosis. These cells promote the generation and migration of fibrotic cells by locally releasing pro-fibrotic factors such as IL-6, which activate renal tubular epithelial cells (RTECs) [120]. Similarly, elevated levels of circulating fibrocytes have been observed in patients with end-stage renal disease (ESRD) who are dependent on hemodialysis [121].

Experimental studies have further elucidated the specific mechanisms by which these cells contribute to renal fibrosis. In the unilateral ureteral obstruction (UUO) mouse model, inflammatory cytokines such as IL-17A and IL-33 have been shown to induce the conversion of circulating fibrocytes into fibroblasts and promote their accumulation in renal tissue [122,123]. Notably, IL-33 activates the STAT3 signaling pathway, driving the differentiation of bone marrow–derived monocytes into fibroblast precursors, thereby exacerbating renal fibrosis [123]. Additionally, Tamibarotene (Am80), by inhibiting the production of IL-17A, reduces fibrocyte accumulation and the expression of related fibrotic markers, indicating that modulation of inflammatory pathways can effectively alleviate renal fibrosis [122]. Furthermore, activation of the erythropoietin (EPO) signaling pathway has been demonstrated to reduce oxidative stress and mitochondrial activity, thereby inhibiting the accumulation of bone marrow–derived fibrocytes and alleviating renal fibrosis [124,125]. These findings suggest that circulating fibrocytes play a significant role in the progression of renal fibrosis through multiple mechanisms.

### 4.6. Conclusions

Across organs, circulating fibrocytes provides a distinctive window into fibrosis because they are measurable in blood, link immune trafficking with ECM-associated programs, and can plausibly participate in injury responses through recruitment, local activation, and paracrine crosstalk. This dual “immune–stromal” positioning makes fibrocytes attractive both as accessible biomarkers and as intervenable nodes in fibrotic cascades that are otherwise dominated by tissue-resident stromal populations [11].

The depth of evidence, however, is uneven across tissues. Lung/airway diseases currently offer the most mature framework, with repeated clinical associations and multiple mechanistic studies converging on chemokine-guided recruitment and activation pathways [32,126]. Cardiac fibrosis shows growing clinical and experimental support, but cellular-origin debates and model-dependent conclusions indicate that fibrocytes are likely to contribute in a stage- and context-specific manner rather than serving as the dominant collagen source [77,84]. Skin fibrosis and scar biology benefit from high accessibility and rich phenotyping opportunities, yet biomarker performance remains variable across disease stages. Liver and kidney sections highlight strong links to inflammatory–fibrotic coupling, but the field still needs more standardized longitudinal human studies to clarify when fibrocytes act mainly as inflammatory amplifiers versus direct ECM contributors [107].

Therapeutically, current data across organs consistently nominate recruitment and activation circuits—notably chemokine axes and upstream inflammatory signaling—as the most tractable strategies, while emphasizing that stage-specific targeting will be essential to avoid disrupting physiological repair.

## 5. Discussion

Circulating fibrocytes originate from hematopoietic stem cells in the bone marrow and migrate to damaged tissues through the bloodstream, playing a crucial role in the repair process [6,29,106]. These cells not only promote local inflammation by secreting cytokines but also contribute to the synthesis of collagen and other extracellular matrix components, thereby exacerbating the fibrotic progression [11,30,127,128]. The function of circulating fibrocytes is closely linked to specific molecular markers such as CD34, CD45, and type I collagen [29]. Currently, the labeling and identification of fibrocytes remain challenging, and their phenotypic characteristics and differentiation pathways require further characterization, particularly the transition from precursor states to mature fibroblasts and the regulatory factors involved in this process [35]. Single-cell sequencing technologies and omics-based research will be powerful tools for elucidating these complex mechanisms in the future.

Although the role of circulating fibrocytes in tissue fibrosis has been extensively studied, further investigation is needed to explore their specific functions and regulatory mechanisms in different types of fibrotic diseases. Current research has primarily focused on the role of circulating fibrocytes in renal and pulmonary fibrosis, but their involvement in other organs, such as the heart, liver, and skin, remains incompletely understood [106,129]. Therefore, future studies should focus on the specific functions of circulating fibrocytes in multi-organ fibrosis and their interactions with different tissue microenvironments, aiming to uncover both the common and distinct mechanisms underlying cross-organ fibrosis.

Currently, research on circulating fibrocytes still faces several challenges. Firstly, despite their key role in fibrosis, the limited number of circulating fibrocytes in peripheral blood, coupled with the lack of effective biomarkers and standardized identification methods, restricts our comprehensive understanding of their biological characteristics and functions. Secondly, targeted therapeutic strategies for circulating fibrocytes are still relatively scarce, and our knowledge of their specific roles and regulatory mechanisms at different stages of the disease remains insufficient, posing challenges to the development of effective anti-fibrotic treatments. Therefore, future research should focus on developing more precise isolation and identification techniques, as well as employing high-throughput screening and gene-targeted editing technologies to better explore the potential of circulating fibrocytes as therapeutic targets and biomarkers. This could provide new strategies for the diagnosis and treatment of fibrotic diseases.

## Figures and Tables

**Table 1:** Comparative phenotypic and functional features of fibrocytes and related cell populations.

Cell Population	Origin/Differentiation	Positive Marker	Key Function
Fibrocyte	Fibrocytes are bone marrow–derived circulating leukocyte-like cells with fibroblast-like properties that can accumulate at sites of tissue injury [6].	Core ECM/hematopoietic markers: CD45; CD34; collagen I (COL1A1/COL1A2); collagen III (COL3A1); fibronectin; vimentin (VIM); Myeloid adhesion-related: CD11b, CD13, CD44 [14]; Chemokine receptors: CCR2/CCR5/CCR7 [15].	Recruitment to injured tissue and participation in repair and fibrotic remodeling programs [11].
Monocyte	Monocytes are bone marrow–derived circulating myeloid cells that can infiltrate tissues and differentiate into macrophages and dendritic cells [16].	CD45, CD14 and CD16 [17].	Innate immune surveillance, cytokine production, tissue infiltration, precursors for macrophage lineages [18].
Macrophage	Tissue-resident and monocyte-derived phagocytes with highly context-dependent activation states [19].	CD45, CD68 and CD163 [2].	Phagocytosis, antigen processing, inflammation resolution, collagen turnover and uptake in remodeling contexts [20].
Fibroblast/Myofibroblast	Tissue-resident stromal cells.	Fibroblasts: COL1A1/2, DCN, LUM; Myofibroblast: ACTA2 [21].	ECM synthesis and remodeling [22].
Mesenchymal stromal cell (MSC)	Multipotent stromal cells (culture-expanded definition) with tri-lineage differentiation capacity and operationally defined by ISCT minimal criteria [23].	CD73, CD90, and CD105 [24].	Paracrine immunomodulation and tri-lineage differentiation capacity in vitro [25].

ISCT: International Society for Cell & Gene Therapy. Markers listed are representative and may vary by species, tissue, disease stage, and assay platform, thus fibrocytes should be considered a heterogeneous and context-dependent population.

## Data Availability

No new data were created or analyzed in this study. Data sharing is not applicable to this article.
